# Molecular communication from bone to skeletal muscle: an overview

**DOI:** 10.3389/fcell.2025.1715009

**Published:** 2025-11-07

**Authors:** Guobin Li, Mingyan Qi, Yuzhen Wang, Shibin Liang, Huiyun Xu

**Affiliations:** 1 College of Life Sciences, Inner Mongolia Agricultural University, Hohhot, Inner Mongolia, China; 2 Inner Mongolia Key Laboratory of Biomanufacturing Techenology, Inner Mongolia Agricultural University, Hohhot, Inner Mongolia, China; 3 Key Laboratory for Space Bioscience and Biotechnology, School of Life Sciences, Northwestern Polytechnical University, Xi’an, Shaanxi, China

**Keywords:** bone, muscle, osteokines, extracellular vesicles, crosstalk, connexin43

## Abstract

The intricate interactions between bone and muscle are central to musculoskeletal health. It was historically assumed that bone and muscle interact through mechanical coupling, that is, skeletal muscles attach to bone and facilitate movement of the bone via muscular contraction. However, recent studies have recognized bone and muscle as endocrine organs, capable of producing and releasing osteokines and extracellular vesicles (EVs) that influence each other’s functions, thereby introducing a novel concept known as “bone-muscle crosstalk”. The influence of muscle on bone has been extensively studied, little has reported regarding the muscle regulation by bone. Emerging studies indicate that the transmission of signaling molecules from bone to muscle is partially mediated by hemichannels and gap junctions formed by connexin 43 (Cx43) in osteoblasts and osteocytes. This review aims to summarize the latest findings on bone-muscle crosstalk, with a particular emphasis on the roles of osteokines and EVs derived from bone. Furthermore, it highlights the channel functions of Cx43 in the release of secretory factors through this crosstalk mechanism. The continued research into bone–muscle crosstalk is expected to identify new therapeutic targets for the twin diseases of osteoporosis and sarcopenia.

## 1 Introduction

In the musculoskeletal system, bone and muscle are closely correlated across the life cycle. They share the common mesodermal precursors during embryogenesis. In case of exercise and disuse, changes in bone and muscle mass are also tightly linked. With aging, there is a simultaneous decline in both bone and muscle mass. Traditionally, this relationship has been understood primarily in terms of mechanical coupling, where bone serve as a scaffold for muscle attachment, and muscle applies load to bone (Zhao et al., 2024). The physical linkage is undoubtedly necessary to support locomotion and the shape/forms of animals. However, the synergy between bone and muscle goes beyond mechanical, as evidenced by the discovery of the endocrine functions of these two tissues (Lee et al., 2007; Kurek et al., 1997; Karsenty and Olson, 2016). Both bone and muscle can produce soluble factors that exert either positive or negative effects on each other (Welc et al., 2025). This intricate reciprocity is central to maintain musculoskeletal health.

Bone is a highly vascularized organ, with osteocytes residing in lacunae that are in close proximity to blood vessels via lacunocanalicular networks. The release of osteokines into the bloodstream appears to be the most prominent mechanism of communication between bone and muscle (Lara-Castillo and Johnson, 2020). Likewise, several myokines produced by muscle also are known to circulate (Zhao et al., 2024). Recent research has identified extracellular vesicles (EVs), which are shed cellular components, as an additional mechanism facilitating crosstalk between bone and muscle (Ma et al., 2023). EVs are lipid bilayer-bound particles that encapsulate various biomolecules, including mRNAs, miRNAs, and proteins, reflecting the cellular state. These shed EVs can exert local effects in an autocrine manner or be transported into circulation to influence distant organs (He C. et al., 2020). Furthermore, due to their anatomical proximity, another potential mechanism of bone-muscle communication involves the diffusion of molecules across the periosteum. An early study by Lai et al. (2014) demonstrated that the semi-permeable periosteum permits the diffusion of molecules smaller than 40 kDa. This suggests that small osteokines can easily reach the adjacent muscle by passive diffusion, and those molecules with greater than 40 kDa are likely to be delivered via the circulation or as EVs cargo.

The adult skeleton predominantly consists of three cell types: osteoblasts, osteoclasts, and osteocytes. Osteocytes, which account for over 90% of the total bone cell population, establish an extensive lacunar-canalicular network facilitating intercellular communication among these cell types (He et al., 2025). One mechanism of cell-cell communication is mediated via gap junctions, which are membrane-spanning channels formed by the docking of two hemichannels (Zhao et al., 2022). In addition to direct intercellular communication through gap junctions, hemichannels facilitate interactions between osteocytes and the pericellular environment. Both types of connexin-based channels exhibit selective permeability, allowing the diffusion of molecules smaller than 1.2 kDa (Zhang et al., 2025). Connexin43 (Cx43) is the most prevalently expressed connexin subtype in osteoblasts and osteocytes. Previous research has demonstrated that Cx43 in bone plays a crucial role in skeletal muscle development, as evidenced by the impaired formation of skeletal muscle in mice lacking Cx43 in osteoblasts/osteocytes (Shen et al., 2015). Our group has recently elucidated the distinct functions of Cx43 hemichannels and gap junctions in osteocytes, which regulate skeletal muscle function (Li et al., 2021; Li et al., 2022). These findings underscore the potential roles of Cx43 in mediating signal transmission from bone to muscle.

Osteoporosis and sarcopenia are major clinical concerns in the aging population, and these two conditions often occur concurrently in many patients. However, the current therapeutic approach for the twin disease mostly targets the one rather than both tissues simultaneously (Kirk et al., 2020a). A treatment paradigm shift may be underway with increasing recognition of the close ties between bone and muscle. Herein, we summarize the latest progress of the role of bone-derived factors and Cx43 in bone-muscle crosstalk. Such knowledge is crucial for the discovery of potential therapeutic targets that may lead to more integrated treatment strategies for the musculoskeletal disorders.

## 2 Bone as an endocrine organ

In addition to provide structural support for the internal organs, bone serves as a substantial reservoir for osteogenic growth factors, such as insulin-like growth factors (IGF_S_), bone morphogenetic proteins (BMPs) and transforming growth factor β (TGFβ), etc (He et al., 2025). These factors play a critical role in the continuous bone remodeling through bone formation by osteoblasts and bone resorption by osteoclasts. Osteocytes, which are embedded within the bone matrix, are also considered indispensable orchestrators of osteoblast and osteoclast functions (Zhang et al., 2025). Recent evidence from multiple research groups supports the notion that bone functions as an endocrine organ. This is primarily due to its highly vascularized nature and its ability to secrete osteokines into the bloodstream, which can influence the function of distant tissues, including muscle.

Of the major cell types in skeleton, osteoblasts constitute only 5% of total bone cells compared to 1% of osteoclasts, and the remaining more than 90% are osteocytes (Zhang and Chen, 2024). Osteocytes, residing within lacunae, extend their dendritic processes to form a lacunocanalicular system that connects with the vasculature in the bone matrix. Considering the substantial mass of osteocytes and their dendritic processes within the skeleton, these cells likely serve as the primary source of circulating factors derived from bone. Various imaging techniques have demonstrated the connectivity between dendritic processes, adjacent osteocytes, and the vasculature. A study conducted by Beno and colleagues (Beno et al., 2006) revealed that the injection of small dyes or molecules, up to 70 kDa in size, into the tail vein of a mouse traverses the lacunocanalicular network within a few minutes. This observation suggests that canalicular fluid permeates into the circulation, allowing osteocyte-secreted factors to potentially influence distant target tissues. To the best of our knowledge, the initial evidence supporting the role of the osteocyte as an endocrine cell was the discovery that fibroblast growth factor 23 (FGF23), which is highly expressed in osteocytes, regulates phosphorus homeostasis in the kidneys (Feng et al., 2006). The list of bone-derived factors continues to expand, with significant examples including osteocalcin, prostaglandin E2 (PGE_2_), insulin-like growth factor 1 (IGF1), receptor activator of nuclear factor kappa β ligand (RANKL), osteoprotegerin (OPG), Wnt proteins, Dickkopf-1 (DKK1), sclerostin, fibroblast growth factor 23 (FGF23), and transforming growth factor β (TGFβ), as elaborated below (Table 1).

**TABLE 1:** Summary of osteokines that regulate muscle physiology.

Osteokines	Regulatory factors	Source	Effects on muscle (ref.)`
Osteocalcin	Vitamin K, exercise-induced IL-6	OB	Promote nutrients uptake and catabolism in muscle during exercise (Mera et al., 2016a) Maintain muscle mass and function in aged mice via protein synthesis pathway (Mera et al., 2016b) Accelerate C2C12 cell proliferation (PI3K/Akt and P38 pathway) and myogenic differentiation (GPRC6A-ERK1/2 pathway) (Liu et al., 2017)
IGF1	Vitamin D, protein and calcium	OB/OCY	Enhance muscle cell proliferation and differentiation (Schiaffino and Mammucari, 2011) Activation of IGF1/Akt pathway leading to muscle hypertrophy (Blaauw et al., 2009)
PGE_2_	COX, PGES	OB/OCY	PGE_2_ mimics the effects of primary osteocyte or MLO-Y4 cells on proliferation (Mo et al., 2015) and differentiation (Mo et al., 2012) of skeletal muscle myoblasts
RANκL/OPG	Exercise-induced IL-6	OC/OCY	Fast-twitch muscle atrophy due to OPG deficiency and high RANκL levels (Hamoudi et al., 2020) RANκL inhibition improves muscle strength in postmenopausal women and *Pparb* ^−/−^ mice model (Bonnet et al., 2023)
FGF23	DMP1, Phex and MEPE	OB/OCY	Induce cardiac hypertrophy but did not alter skeletal muscle function (Faul et al., 2011; Avin et al., 2018)
TGFβ	Tumor-induced osteolysis	OB	Reduce muscle force production under pathological conditions (Waning et al., 2015)
Wnt3a	Fluid flow shear stress	OCY	Promote C2C12 cell differentiation (Huang et al., 2017)
Sclerostin	PTH	OCY	Sclerostin ablation increase lean body mass in aged animals (Kim et al., 2017)
Unknown	Fluid flow shear stress	OCY	Increase muscle size and contractile force with age in *Mbtps1* cKO mouse model (Gorski et al., 2016) Murine osteocytic cell line (Ocy454) secretome inhibits C2C12 cell differentiation (Wood et al., 2017)
VEGF	Hypoxia	BMSC	Improve muscle regeneration in pathological conditions (Zhou et al., 2015)

## 3 Bone-muscle crosstalk and involvement of bone factors

**FIGURE 1:**
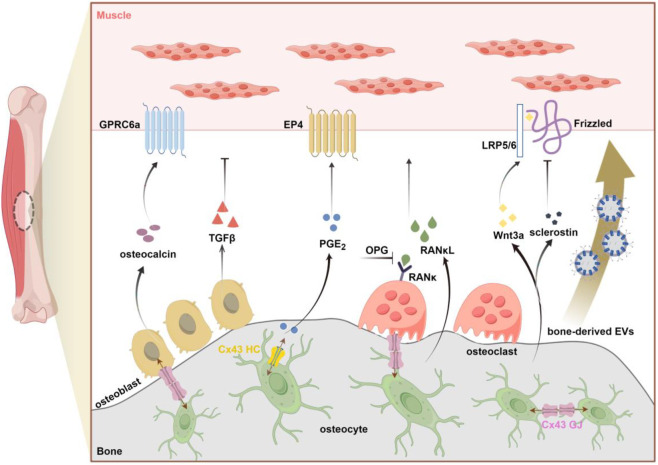
Schematic overview of bone-derived factors (osteokines and EV_S_) involved in bone-to-muscle communication (by figdraw.com). Primary factors released from bone that affect muscle including: osteocalcin, TGFβ (Transforming growth factorβ), PGE_2_ (Prostaglandin E2), RANκL (Receptor Activator of Nuclear Factor Kappa β Ligand), OPG (osteoprotegerin), Wnt3a, sclerostin. Emerging evidence also supports the potential role of bone-derived EVs in signal transmission from bone to muscle. As the key gateway to the passasge of signaling molecules, Cx43-formed GJ and HC in osteocytes can regulate bone-muscle crosstalk through the release of small molecules such as PGE_2_. GPRC6a, G protein-coupled receptor; EP4, E-type prostanoid receptor4; LRP5/6, lipoprotein receptor-related protein 5/6; Cx43, connexin43; GJ, gap junction; HC, hemichannel.

The intricate relationship between bone and muscle is established during fetal development, as both tissues originate from common progenitor cells and undergo organogenesis regulated by a complex gene network (Dong et al., 2024). Historically, the emphasis on the mechanical coupling between these two tissues may have stemmed from embryological studies. In the developing embryo, muscle forces significantly influence skeletal growth and bone morphology, while skeletal adaptations in early postnatal life are primarily driven by changes in mechanical stimuli (Deng et al., 2024). However, a more comprehensive understanding of bone-muscle interaction beyond mechanical coupling to include a wide array of signaling factors exchanged between the two tissues. Also, the consequences of bone-to-muscle signaling mainly include alterations in skeletal muscle mass and muscle function (Figure 1).

### 3.1 Role of bone-derived osteokines

#### 3.1.1 Osteocalcin

Osteocalcin, also referred to as carboxyglutamic acid or BGLAP, is a protein secreted by mature osteoblasts and osteocytes. Osteocalcin undergoes post-translational modification at three specific glutamate residues (in positions 17, 21, and 24), by γ-glutamyl carboxylase with vitamin K as a cofactor (Kirk et al., 2025). Due to the high affinity of osteocalcin for hydroxyapatite crystals following its γ-carboxylation, most secreted osteocalcin is deposited in the mineralized bone matrix (Battafarano et al., 2020). It can be released into the circulation through decarboxylation at low pH levels. Osteocalcin has been shown to affect distant adipocytes and pancreatic β-cells by binding to the Gprc6a receptor. Beyond its role in the regulation of energy metabolism (Lee et al., 2007), glucose metabolism (Kanazawa, 2015), and ectopic calcification (Bonewald, 2019) in rodent models, osteocalcin also affects muscle physiology. The study by Karsenty and colleagues demonstrated a significant reduction in muscle mass in Gprc6a^−/−^ mice. Conversely, Esp^−/−^ mice, which lack a phosphatase that inhibits osteocalcin function, exhibit increased muscle mass. Furthermore, osteocalcin supplementation enhances exercise capacity in young mice and mitigates age-related declines in muscle strength. Aerobic exercise increases circulating osteocalcin levels and osteocalcin signaling in muscle tissue, leading to the secretion of the myokine IL-6 (Mera et al., 2016a). The mechanism by which exercise affect osteocalcin and interleukin-6 (IL-6) involves the exercise-induced release of the myokine IL-6, which acts on osteoblasts. This interaction results in increased secretion of receptor activator of nuclear factor kappa-Β ligand (RANκL) by osteoclasts, facilitating the remodeling of the bone matrix. Consequently, osteocalcin is liberated from the bone matrix into the circulation, where it reaches muscle tissue and binds to its receptor, Gprc6a, thereby modulating muscle function (Chowdhury et al., 2020). These findings provide robust evidence supporting the beneficial role of osteocalcin in the regulation of muscle mass and function.

#### 3.1.2 Prostaglandin E2 (PGE_2_)

Prostaglandin E2 (PGE_2_) is an eicosanoid compound derived from arachidonic acid that can be generated by a reaction catalyzed via cyclooxygenase (COX) and terminal PGE_2_ synthases (PGES) (Cheng et al., 2021). This soluble factor can be released by bone cells and participates in the regulation of various physiological responses, including inflammation, tissue repair, and regeneration. A recent study by Palla et al. demonstrated the beneficial roles of PGE_2_ signaling in the rejuvenation of aged muscle mass and strength (Palla et al., 2021). The study found that elevated levels of 15-hydroxyprostaglandin dehydrogenase (15-PGDH), an enzyme responsible for the degradation of PGE_2_, in aged muscle contribute to muscle atrophy and decreased muscle strength. Moreover, the physiological restoration of PGE_2_ levels through the inhibition of 15-PGDH was shown to enhance mitochondrial function, thereby increasing muscle mass and function. Notably, a comparison with muscle cells revealed that osteocytes produce PGE_2_ at levels 100 to 1000 times higher (Welc et al., 2025). This substantial production of PGE_2_ by osteocytes plays a crucial role in supporting muscle regeneration and the repair of injured muscle tissue (Ho et al., 2017). Moreover, multiple investigations conducted by Brotto’s research group have demonstrated that PGE_2_ serves as a potent stimulator of myogenesis and enhances primary muscle function in *ex vivo* studies (Mo et al., 2015; Mo et al., 2012). An earlier *in vivo* investigation by Wang et al. (Wang et al., 2005) revealed that PGE_2_, released from mechanically stimulated osteocytes, was detectable in the circulatory system. These findings suggest that bone, particularly osteocytes, can modulate muscle function through the secretion of PGE_2_. Nonetheless, the mechanism by which PGE_2_ produced by osteocytes reaches muscle cells remains unclear, given its short half-life in circulation.

#### 3.1.3 Insulin-like growth factor 1 (IGF1)

Insulin-like Growth Factor 1 (IGF-1) is recognized as a crucial anabolic factor in both embryonic and postnatal skeletal muscle development. IGF-1, produced by osteoblasts, may be either secreted freely or deposited into the bone matrix, from which it is released through osteoclast-mediated bone resorption (Wildemann et al., 2007). Osteocyte-derived IGF-1 signaling serves as a critical component of mechanotransduction in bone. The upregulation of IGF-1 expression in osteocytes represents one of the earliest responses of bone to mechanical loading. In addition, nutritional factors including vitamin D, protein and calcium can also upregulate IGF-1 to synergistically regulate the muscle anabolism (Kirk et al., 2020b). As a key regulator of muscle mass during development, IGF-1 has been demonstrated to enhance both the proliferation and differentiation of myogenic cells (Schiaffino and Mammucari, 2011). In adult skeletal muscle, the activation of Akt, a downstream effector of IGF-1 signaling, induces a significant hypertrophic response, characterized by an increase in absolute force without alterations in specific force (Blaauw et al., 2009; Regan et al., 2017). Similarly, Bone Morphogenetic Protein 2 (BMP2) signaling has been shown to sustain and promote adult muscle mass. Notably, BMP2-induced muscle hypertrophy is largely reflected by an increase in absolute muscle force, with specific muscle force remaining unchanged or slightly reduced compared to control mice (Winbanks et al., 2013; Sartori et al., 2014). However, the role of IGF-1 in bone-muscle communication warrants further investigation.

#### 3.1.4 Receptor activator of nuclear factor kappa β ligand (RANκL)

Osteocytes are the primary source of RANκL, an osteokine that plays a crucial role in osteoclast activity and formation. The deletion of RANκL results in significant bone loss, including tooth loss and the absence of osteoclasts in mice (Kong et al., 1999). The receptor for RANκL, known as RANκ, is expressed in both osteoclasts and fully differentiated myotubes. The interaction between RANκL and RANκ induces osteoclast activation and osteoclastogenesis via NF-κB signaling. Activation of RANκ can inhibit myogenic differentiation and activate the ubiquitin–proteasome system, ultimately leading to muscle atrophy (Kirk et al., 2025; Langen et al., 2001). Osteoprotegerin (OPG), a decoy receptor for RANκL, exerts an inhibitory effect on osteoclast differentiation. A study on glucocorticoid-induced osteoporotic rats showed that treadmill training significantly decreased RANκL expression and increased OPG levels, suggesting that the RANκ/RANκL/OPG signaling is modulated by exercise (Pichler et al., 2013). In addition, elevated circulating IL-6 levels during exercise can also signal to osteoblasts to produce RANκL. Recent evidence suggests a role for RANκL in bone-muscle crosstalk. The OPG knockout mouse exhibits reduced bone mass and fast-twitch muscle atrophy due to elevated RANκL levels (Hamoudi et al., 2020). Furthermore, improvements in bone biomechanical properties and fast-twitch muscle mass have been observed with the administration of an anti-RANκL antibody. In 2024, Gostage and colleagues (Gostage et al., 2024) demonstrated that the ablation of RANκL (RANκL^−/−^) or OPG (Opg^−/−^) in mice resulted in deleterious effects on both bone and muscle. Conversely, beneficial effects were observed when these mice were treated with anti-RANκL or OPG-Fc. Clinical data further suggest that a three-year treatment regimen with the anti-RANκL antibody denosumab can enhance lean muscle mass and strength in women (Bonnet et al., 2023). Notably, the effects of anti-RANκL therapy appear to be specifically targeted towards fast-twitch skeletal muscle. Consequently, the RANκ-RANκL-OPG pathway is regarded as a therapeutic target for osteoporosis and sarcopenia.

#### 3.1.5 Regulators of Wnt/β-catenin pathway

The Wnt/β-catenin signaling pathway plays an essential role in regulating the differentiation of bone marrow mesenchymal stem cells into osteoblasts during embryonic development, maintaining bone homeostasis during postnatal growth, and facilitating bone accrual in response to mechanical loading (Huang et al., 2017). Various components of this pathway serve as key regulators, enabling osteocytes to transmit mechanical loading signals to cells on the bone surface. Mechanical loading can activate the Wnt/β-catenin pathway through its interaction with the prostaglandin pathway, resulting in an increase in positive regulators of bone formation, such as Wnt proteins, and a decrease in negative regulators, including Dkk-1 and sclerostin (Kitase et al., 2010). Although these factors primarily originate from bone and exert local effects, such as Wnt 3a and sclerostin, are also detectable in serum, suggesting their potential role in bone-muscle crosstalk regulation. Wnt 3a is secreted by osteocytes, and its expression level is significantly elevated in MLO-Y4 cells subjected to fluid flow shear stress (Huang et al., 2017). Research conducted by Brotto’s group has demonstrated that osteocyte-derived Wnt3a promotes the myogenic differentiation of C2C12 mouse myoblasts and human muscle cells by upregulating myogenin and MyoD. Conversely, sclerostin acts as an inhibitor of Wnt 3a, thereby hindering myoblast differentiation (Huang et al., 2017; Jähn et al., 2012). Despite the current understanding of the roles of Wnt proteins and sclerostin in bone-muscle crosstalk, *in vivo* evidence remains insufficient. In addition to the skeleton, sclerostin is also found in serum and its circulating levels associated with whole-body metabolism, are affected by sex hormones, and respond to intermittent parathyroid hormone (PTH) (Chen et al., 2022). Notably, a study utilizing a breast cancer mouse model demonstrated that the administration of an anti-sclerostin antibody effectively prevented bone destruction and enhanced skeletal muscle function, in contrast to the outcomes observed in vehicle-treated mice (Hesse et al., 2019). It has been established that Dkk-1 in bone is predominantly secreted by osteoblasts rather than osteocytes (Ke et al., 2012). Mice deficient in Dkk-1 exhibit high bone mass despite elevated levels of circulating sclerostin. However, the potential impact of Dkk-1 on muscle remains unclear. These findings prompt several unresolved questions, such as the mechanism by which osteocyte-derived Wnt3a affects muscle function—whether through an endocrine pathway, via extracellular vesicles, or through an alternative mechanism.

#### 3.1.6 Fibroblast growth factor 23(FGF23)

Contrary to the hypertrophic response induced by bone-derived factors, certain osteokines have been demonstrated to adversely affect muscle mass and function. Since its identification in 2000, fibroblast growth factor 23 (FGF23) has been recognized as an osteocyte-produced hormone that plays a crucial role in renal phosphate handling and the synthesis of 1,25-dihydroxyvitamin D (1,25D), the most biologically active form of vitamin D (Shimada et al., 2004; Riminucci et al., 2003). The secretion of FGF23 is co-regulated by other osteocyte-derived factors, including dentin matrix protein 1 (Dmp1), phosphate-regulating neutral endopeptidase X-linked (Phex), and matrix extracellular phosphoglycoprotein (MEPE) (Delgado-Calle and Bellido, 2022). In the absence of Phex or Dmp1, an increase in systemic FGF23 levels in osteocytes results in enhanced phosphate excretion by the kidneys, leading to conditions such as rickets and osteomalacia. Recent studies suggest that FGF23 plays a critical role in regulating phosphate homeostasis in response to exercise, with osteocyte responsiveness to the exercise-induced myokine β-aminoisobutyric acid (BAIBA) influencing this process during aging, as reviewed by Welc and colleagues (Welc et al., 2025).

The activation of canonical FGF23 signaling necessitates interaction with the essential co-receptor α-klotho, whereas non-canonical FGF23 signaling operates independently of α-klotho (Kirk et al., 2025). Both FGF23 and α-klotho have been demonstrated to inhibit the myogenic differentiation of cultured human skeletal muscle cells by downregulating IGF1 signaling. Furthermore, FGF23 induces premature senescence in mesenchymal stem cells (MSCs) derived from human skeletal muscle via the p53-p21 pathway, independently of α-klotho, without impacting satellite cell function (Sato et al., 2016). In a rickets/osteomalacia model, administration of an FGF23 neutralizing antibody in mice resulted in elevated serum phosphate levels and enhanced muscle function (Regan et al., 2017; Waning and Guise, 2014). Additionally, skeletal muscle function was impaired in Dmp1-deficient mice, although cardiac force production remained unaffected (Wacker et al., 2016). These findings suggest that osteocyte-derived FGF23 may serve as a potential mediator in bone-to-muscle communication, warranting further investigation into its precise role.

#### 3.1.7 Transforming growth factorβ (TGFβ)

Transforming Growth Factor Beta (TGFβ) is produced by a variety of tissues throughout the body, with the skeletal system serving as the predominant source. This cytokine is primarily synthesized by bone-forming osteoblasts and is stored in the mineralized matrix in a latent form. The activation and release of TGFβ occur in response to low pH conditions during osteoclast-mediated bone resorption or mechanical stretching (Wang et al., 2022). Beyond its interactions with osteoclasts and osteoblasts, TGFβ also plays a critical role in remodeling the osteocyte lacunocanalicular network. The deletion of TGFβ in osteocytes has been associated with increased bone fragility (Schurman et al., 2021). A study by Waning and colleagues (Waning et al., 2015) has demonstrated that TGFβ is integral to bone-muscle communication. Specifically, bone degradation resulting from cancer metastasis leads to elevated TGFβ release from the bone matrix, which subsequently contributes to muscle weakness by impairing calcium-induced muscle force production. Interventions utilizing the bone-targeting bisphosphonate zoledronic acid or the TGFβ receptor I kinase inhibitor SD-208, aimed at inhibiting TGFβ signaling, have shown promise in ameliorating skeletal muscle wasting and weakness. These findings underscore the detrimental effects of osteoclast-mediated TGFβ release on skeletal muscle health.

#### 3.1.8 Vascular endothelial growth factor (VEGF)

Vascular endothelial growth factor (VEGF), produced by bone, is an endothelial cell survival factor that coordinates the processes of angiogenesis and osteogenesis. It plays a central role in bone homeostasis, repair, and the pathobiological processes affecting these functions (Chen et al., 2023). Bone marrow mesenchymal stem cells (BMSCs) function as progenitor cells within the bone marrow niche and have the capacity to differentiate into various cell types, including osteoblasts and myoblasts (Han et al., 2025; Zhu et al., 2021). The unique ability of BMSCs to modulate the immune system and facilitate tissue repair distinguishes them from other stem cell types, indicating that BMSCs may be ideal candidates for use in tissue engineering and regenerative medicine. Notably, BMSCs derived from patients with amyotrophic lateral sclerosis (ALS) exhibit reduced stem cell capacity and produce fewer trophic factors, with these deficiencies correlating with disease progression (Zhou et al., 2015). Research has demonstrated that the paracrine release of vascular endothelial growth factor (VEGF) by BMSCs in the bone marrow enhances muscle regeneration (Sassoli et al., 2012). These findings suggest a potential role for BMSC-derived VEGF in bone-muscle crosstalk. However, the molecular mechanisms underlying this potential interaction between muscle and bone remain largely unexplored.

### 3.2 Role of bone-derived extracellular vesicles

In addition to the conventional factors previously discussed, extracellular vesicles (EVs) are increasingly recognized as novel contributors to bone-muscle crosstalk (Figure 1). EVs, a class of membrane-bound particles released by nearly all cell types, convey information in the form of proteins, mRNAs, and miRNAs (Kirk et al., 2025; Van Niel et al., 2018). These vesicles can be categorized based on their size, synthesis, and secretion mechanisms. Currently, the most extensively characterized EVs are exosomes (20–140 nm) and microvesicles (100 nm-1 μm) (Huang et al., 2022). EVs, along with their molecular cargo, traverse the circulatory system and interact with distant target cells, influencing their differentiation and/or function. The interactions between EVs and their target cells primarily occur through mechanisms such as endocytosis, receptor-ligand binding, fusion with the plasma membrane, and antigen presentation (Kirk et al., 2025). In the context of bone-muscle crosstalk, EVs may play a role by facilitating the exchange of myokines, osteokines, and organelles (Ma et al., 2023; Murray and Krasnodembskaya, 2019).

At present, specific cell surface markers for the identification or enrichment of extracellular vesicles (EVs) derived from bone cells, such as osteoblasts, osteoclasts, and osteocytes, remain unidentified. While E11/gp38 and Phex have been proposed as potential markers for osteocyte-derived EVs, they lack specificity. The use of DMP1 and sclerostin as surface markers for osteocyte-derived EVs is contentious, despite their expression in early-stage and mature osteocytes, respectively. Alkaline phosphatase (ALP) may serve as an identifier for osteoblast-derived EVs, whereas potential surface markers for osteoclast-derived EVs include DC-STAMP, OSCAR, and the calcitonin receptor. Further research is necessary to delineate the markers that can effectively distinguish subpopulations of circulating EVs originating from various bone cell types (Qin and Dallas, 2019).

Previous research has identified a subset of differentially expressed extracellular vesicle microRNAs (EV-miRNAs) between young and aging BMSCs. Among these, muscle-targeting miRNAs such as miR-24, miR-328-3p, miR-365, and miR-374 are downregulated, whereas miR-15b, miR-17, miR-20a, miR-186, miR-221, miR-31a-5p, and miR-99b are upregulated (He C. et al., 2020). Sun et al. (2008) demonstrated that the absence of miR-24 inhibits myogenic differentiation in C2C12 cells, while its ectopic expression counteracts the anti-myogenic effects induced by TGFβ1. Additionally, the pro-osteogenic miRNAs miR-365 and miR-374 have been reported to promote cardiomyocyte hypertrophy by inhibiting autophagy through the Skp-2-mTOR (Wu et al., 2017) and VEGF (Lee et al., 2017) pathways, respectively. A study involving 93 elderly patients clinically diagnosed with sarcopenia found that the circulating levels of miR-328 were significantly lower in individuals with sarcopenia compared to those without the condition (He N. et al., 2020), potentially due to miR-328's activation of the Wnt/β-catenin pathway via targeting axin-1 (Liu D. et al., 2018). Moreover, miR-328 is highly expressed in apoptotic bodies derived from BMSCs, which exhibit impaired osteogenic differentiation and self-renewal in an apoptotic-deficient mouse model (MRL/lpr-Casp3^−/−^) (Liu D. et al., 2018). These findings suggest that miR-328 may play a role in mediating bone-muscle crosstalk during aging.

The overexpression of miR-15b has been documented to inhibit myoblast differentiation via SET-domain containing 3 (SETD3), a methyltransferase implicated in the regulation of myogenesis (Zhao et al., 2019). Research has indicated that members of the miR-17–92 cluster, specifically miR-17 and miR-20a, can enhance the proliferation of C2C12 myoblasts while concurrently inhibiting myogenic differentiation (Qiu et al., 2016). Additionally, the knockdown of miR-17 has been shown to positively affect the microstructure of trabecular bone (Fang et al., 2016). However, it remains unclear whether miR-17 and miR-20a function as a cluster encapsulated within extracellular vesicles (EVs) to impact the phenotypes of bone and muscle during aging. As a negative regulator of bone formation, the ectopic overexpression of miR-221 has been demonstrated to impede myotube formation (Gan et al., 2020; Liu B. et al., 2018). *In vitro* studies have revealed that miR-186 exerts an inhibitory effect on myogenin-dependent differentiation (Antoniou et al., 2014). miR-31a-5p is upregulated in extracellular vesicles derived from aged bone marrow stromal cells (BMSCs), promoting osteoclastogenesis, adipogenesis, and bone resorption (Xu et al., 2018). Additionally, the age-related increase in miR-31a-5p inhibits the dystrophin response to mechanical loading, thereby heightening muscle susceptibility to disuse-induced injury (Hughes et al., 2018). In primary human myotubes, the overexpression of miR-99b results in decreased protein synthesis by inhibiting the regulatory-associated protein of mTOR (RPTOR) (Zacharewicz et al., 2020). As for bone, Franceschetti et al. (Franceschetti et al., 2014) demonstrated that the inhibition of miR-99b reduces both the size and number of osteoclasts during osteoclastogenesis. These findings suggest that miR-99b may serve as a novel therapeutic target for addressing osteo-sarcopenia in the elderly.

## 4 The channel functions of Cx43 in bone-muscle communication

Connexins (Cx) are expressed in bone and skeletal muscle, with Cx43 being the most prevalent connexin in these tissues (Deng et al., 2022). Structurally, Cx43 consists of four transmembrane domains, two extracellular loops, one intracellular loop, and cytosolic amino-terminal and carboxy-terminal regions (Plotkin et al., 2017). Connexons are formed through the oligomerization of six connexin proteins. These structures, also known as hemichannels, facilitate communication between bone cells and the extracellular environment. When two hemichannels from adjacent cells dock together, they form gap junction channels that enable intercellular communication. These connexin-based channels are selectively permeable to molecules smaller than 1.2 kDa due to their relatively low substrate selectivity. Substantial evidence highlights the critical roles of Cx43 in the development and maintenance of bone and skeletal muscle.

Since the proposal in 2006 that bone functions as an endocrine organ, there has been a growing interest in elucidating the crosstalk between bone and muscle. In this context, a seminal study by Shen et al. (Shen et al., 2015) was the first to employ *in vivo* experiments to elucidate the pivotal role of connexin 43 (Cx43) in modulating bone-muscle communication. Mice with a targeted deletion of Cx43, achieved through the expression of Cre recombinase in osteoblast progenitors (Col α1-Cre; Cx43^fl/fl^), exhibited impaired muscle development, characterized by a significant reduction in both muscle mass and grip strength. This reduction in muscle mass contributes to a lower overall body weight, a phenomenon not observed in Cx43-deficient mice with deletions in mature osteoblasts/osteocytes or in osteocytes alone (Plotkin et al., 2008; Bivi et al., 2012). Notably, the administration of the bone-derived factor undercarboxylated osteocalcin (glu-OC) partially ameliorates the compromised muscle function. The observed phenotypes in mice suggest that Cx43 expression in osteoblast precursors is crucial for optimal skeletal muscle development and underscores the significant role of osteocalcin in bone-muscle communication. Similar to connexins, pannexins in bone cells also form hemichannels within the cell membrane; however, there is currently no evidence supporting their ability to form gap junction channels that connect adjacent cells (Plotkin et al., 2017; Luo et al., 2024). Pannexin1 (Panx1) is the predominant pannexin subtype expressed across all bone cells, and female mice with osteocytic Panx1 deletion (Panx1Δot) exhibit increased muscle mass without alterations in muscle strength (Aguilar-Perez et al., 2019). However, the roles of pannexin channels in bone-muscle crosstalk remain insufficiently explored.

The knockout of Cx43 in osteoblasts and osteocytes results in impaired muscle development; however, Cx43 deficiency concurrently disrupts the function of both gap junctions and hemichannels. Consequently, it remains unclear which of these channel types is responsible for the observed muscle phenotypes. To address this, our research group has previously developed two transgenic mouse models to investigate the distinct roles of Cx43 hemichannels and gap junctions specifically in osteocytes. Utilizing a 10 kb-DMP1 promoter, the transgenic mice, R76W and Δ130-136, overexpress dominant-negative Cx43 mutants in osteocytes (Xu et al., 2015). In the R76W point mutant model, where the amino acid arginine-76 (R) is substituted with tyrosine (W), Cx43 is able to form functional hemichannels but not gap junctions. Conversely, in the Δ130–136 mutant, characterized by the deletion of amino acids at positions 130–136, Cx43 is unable to form either hemichannels or gap junctions. The fast-twitch muscle phenotypes observed in Δ130–136 mice are analogous to those in osteoblast/osteocyte-specific Cx43 conditional knockout (cKO) mice driven by the 2.3-kb Col1a1 promoter (Shen et al., 2015), indicating that Cx43 deficiency in osteocytes impairs hemichannel function, thereby affecting muscle development. In contrast, the obstruction of Cx43 gap junctions results in diminished muscle contractile force and myogenesis. Relative to wild-type (WT) mice, these two transgenic mouse models exhibited reduced levels of prostaglandin E2 (PGE_2_) in both the circulatory system and primary osteocyte-conditioned media (Li et al., 2021). As previously discussed, PGE_2_ released by osteocytes via Cx43 hemichannels has been demonstrated to facilitate myogenic differentiation and enhance muscle function. In alignment with these observations, our recent investigation has shown that the intraperitoneal administration of PGE_2_ partially ameliorates the deficits in muscle mass and function observed in Cx43 transgenic mice. Furthermore, the diminished PGE_2_ levels in osteocytes, resulting from compromised Cx43 hemichannels, contribute to increased collagen deposition in aged skeletal muscle, a process mediated by the activation of the TGFβ/Smad2/3 signaling pathway (Li et al., 2022). In summary, Cx43 hemichannels and PGE_2_ in osteocytes are likely to play a pivotal role in the communication between bone and muscle (Figure 1).

## 5 Conclusion and future directions

Recent advances in our understanding of bone-muscle crosstalk have been significant. This progress can be attributed to the identification of bone as an endocrine organ and the discovery of osteokines, cytokines released by bone that facilitate communication with skeletal muscle. In this review, we examine the roles of bone-derived factors and the potential mechanisms underlying Cx43-mediated crosstalk between bone and muscle. However, there are still many unanswered questions in the field.

Firstly, more unknown bone factors remain to be further identified. An example is that mice with osteocyte-specific deletion of Mbtps1, a membrane-bound transcription factor, exhibit an age-related increase in muscle mass and contractile force in the slow-twitch soleus muscle (SOL) (Gorski et al., 2016). This indicates that osteocytes are likely to produce an unidentified muscle factor that is negatively regulated with aging, which is associated with the production of other negative factors such as RANκL and sclerostin. Furthermore, Connexin 43 (Cx43), as a fundamental component of functionally specific gap junctions and hemichannels, facilitates cellular communication through the release of small molecules. Recently, Cx43 has also garnered attention for its channel-independent cellular regulatory and signaling functions mediated through its specialized C-terminus. Several studies have demonstrated that Cx43 acts as a scaffold protein, and its interactions with cytoskeletal proteins play a crucial role in regulating cell growth, differentiation, and migration (Strauss and Gourdie, 2020; Casanellas et al., 2022). Nevertheless, the extent to which the non-channel functions of Cx43 regulate bone-muscle crosstalk remains largely unclear, presenting potential avenues for future research. Additionally, the burgeoning interest in the roles of extracellular vesicles (EVs) in cellular communication has gained significant attention. EVs encapsulate a diverse array of bioactive molecules, such as proteins, lipids, and nucleic acids, which facilitate the exchange of information between both local and distant organs. Notably, the presence of Cx43 in EVs has been documented, where it enhances EV-cell communication (Xiong et al., 2024). The relationship between Cx43 and extracellular vesicles (EVs) in bone-muscle crosstalk warrants further investigation. Elucidating the complex regulatory networks that mediate the interaction between bone and muscle is crucial to develop small molecule drugs that target Cx43 hemichannels or EVs preparations loaded with bone-derived miR-328 for the combined treatment of osteosarcopenia.

## Glossary

EVs: Extracellular Vesicles
Cx43: Connexin43
IGFS: Insulin-like Growth Factors
BMPs: Bone Morphogenetic Proteins
TGFβ: Transforming Growth Factor β
FGF23: Fibroblast Growth Factor 23
IGF1: Insulin-like Growth Factor 1
RANκL: Receptor Activator of Nuclear Factor kappa β Ligand
OPG: Osteoprotegerin
Dkk1: Dickkopf-1
OB: Oteoblast
OCY: Osteocyte
OC: Osteoclast
BGLAP: Bone γ -Carboxyglutamic Acid Protein
GPRC6a: G Protein-Coupled Receptor 6a
IL-6: Interleukin-6
15-PGDH: 15-Hydroxyprostaglandin Dehydrogenase
BMP2: Bone Morphogenetic Protein2
RANκ: Receptor Activator of Nuclear Factor Kappa β
MyoD: Myogenic Differentiation
DMP1: Dentin Matrix Protein 1
PHEX: Phosphate-regulating neutral Endopeptidase X-linked
MEPE: Matrix Extracellular Phosphoglycoprotein
BAIBA: β-aminoisobutyric Acid
MSCs: Mesenchymal Stem Cells
DC-STAMP: Dendritic cell-specific transmembrane protei
OSCAR: Osteoclast Associated Receptor
BMSCs: Bone Mesenchymal Stem Cells
miRNA: Micro RNA
SKP2: S-phase Kinase-associated Protein 2
mTOR: Mammalian Target of Rapamycin
VEGF: Vascular Endothelial Growth Factor
SETD3: SET-domain containing 3
RPTOR: Regulatory-associated protein of mTOR
GJ: Gap Junction
HC: Hemichannel
EP4: E-type Prostanoid Receptor 4
LRP5/6: Lipoprotein Receptor-related Protein 5/6
glu-OC: Undercarboxylated Osteocalcin
Panx1: Pannexin1
WT: Wild type
cKO: Conditional knockout
α-klotho: α-Klotho Protein
BMSCs: Bone marrow mesenchymal stem cells
COX: Cyclooxygenase
PGES: PGE synthases
PTH: Parathyroid hormone
VEGF: Vascular endothelial growth factor.
